# Influence of the microbiota on the effectiveness and toxicity of oncological therapies, with a focus on chemotherapy

**DOI:** 10.3389/pore.2023.1611300

**Published:** 2023-08-02

**Authors:** Massimiliano Cazzaniga, Giordano Bruno Zonzini, Francesco Di Pierro, Chiara Maria Palazzi, Marco Cardinali, Alexander Bertuccioli

**Affiliations:** ^1^ Scientific & Research Department, Velleja Research, Milano, Italy; ^2^ Department of Biomolecular Sciences, University of Urbino Carlo Bo, Urbino, Italy; ^3^ Department of Medicine and Surgery, University of Insurbia, Varese, Italy; ^4^ Associazione Italiana Fitness e Medicina, Ravenna, Italy; ^5^ Department of Internal Medicine, Infermi Hospital, Azienda Unità Sanitaria Locale Romagna, Rimini, Italy

**Keywords:** microbiota, chemotherapy, oncology, toxicity, microbiome

## Abstract

Recent studies have highlighted a possible correlation between microbiota composition and the pathogenesis of various oncological diseases. Also, many bacterial groups are now directly or indirectly associated with the capability of stimulating or inhibiting carcinogenic pathways. However, little is known about the importance and impact of microbiota patterns related to the efficacy and toxicity of cancer treatments. We have recently begun to understand how oncological therapies and the microbiota are closely interconnected and could influence each other. Chemotherapy effectiveness, for example, appears to be strongly influenced by the presence of some microorganisms capable of modulating the pharmacokinetics and pharmacodynamics of the compounds used, thus varying the real response and therefore the efficacy of the oncological treatment. Similarly, chemotherapeutic agents can modulate the microbiota with variations that could facilitate or avoid the onset of important side effects. This finding has or could have considerable relevance as it is possible that our ability to modulate and modify the microbial structure before, during, and after treatment could influence all the clinical parameters related to pharmacological treatments and, eventually, the prognosis of the disease.

## Introduction

The microbiota, defined as the set of microorganisms that is hosted in each human being, is now considered a fundamental element in human pathophysiology, being able—depending on its composition and structure—to protect us from certain diseases or, on the contrary, contribute to their onset [1]. Oncological pathologies are no exception to this well-established rule and some bacterial groups could be directly or indirectly related to the onset of numerous tumors [2]. A clear example of this relationship is the link between some vaginal community state types (CSTs) and cervical cancer, or the presence of *Fusobacterium nucleatum* and colorectal cancer [3]. Despite the growing amount of information in this field, little is still known about the impact of the microbiota on the progression and prognosis of oncological diseases and, in particular, little consideration is given to the effect that microbiota composition has on the various conventional anti-tumor therapies. Instead, a strong influence arises both in terms of efficacy and toxicity, therefore influencing the prognosis of the disease as well. Chemotherapy above all seems to be affected by these mechanisms and in this review, we will try to highlight the main consequences of this relationship and some of the therapeutic possibilities that we already have available to modulate this effect.

## Chemotherapy and microbiota relationship

**FIGURE 1:**
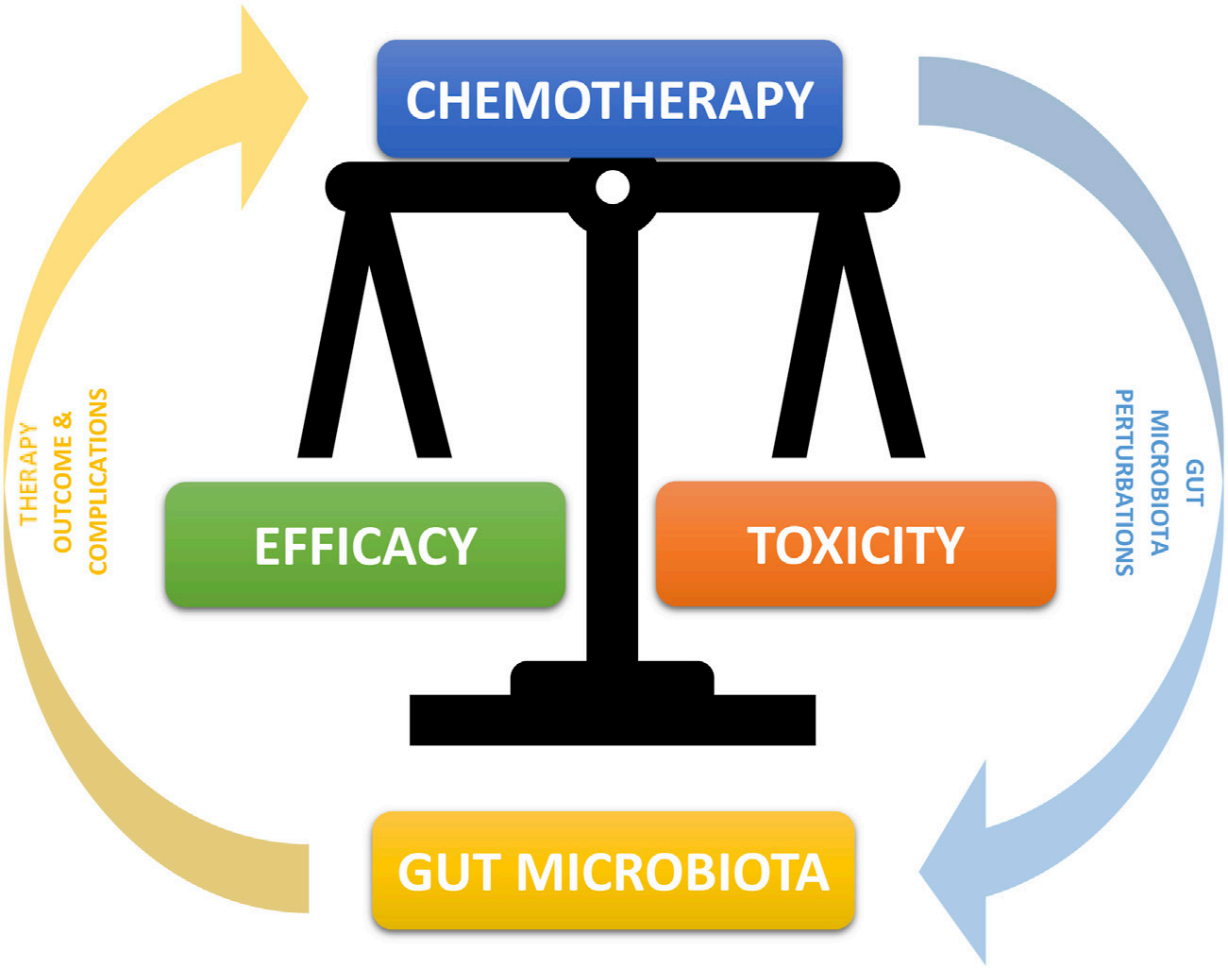
Bidirectional Interconnection between microbiota and chemotherapy. The microbiota can through many mechanisms alter and modify the chemotherapy compound by increasing or decreasing its efficacy. At the same time, chemotherapeutic compounds have an effect of alteration of the microbiota, in particular with a decrease in some symbiotic bacterial populations and an increase in potentially pathological ones, with the consequent onset of greater drug toxicity and a higher incidence of concomitant pathologies.

To date, the choice of chemotherapy treatment, the dosage, the method of administration, and other characteristics of the therapeutic path are substantially determined by well-defined protocols built on the basis of the pharmacokinetic and pharmacodynamic properties of the compound to be used, obviously associated with the patient’s personal characteristics such as their pathology and their clinical and anthropometric parameters [4]. Numerous emerging data, however, highlight the emerging necessity to know a further characteristic of the patient, namely their microbial composition. A very authoritative review on this subject published in 2017 [5] declares that “clinical studies suggests that the composition of the microbiota regulates the efficacy of anticancer therapy and that targeting the microbiota may improve drug efficacy and/or adverse effects.” This work also highlights the fact that the microbiota impacts chemotherapy both when taken orally and parenterally and introduces a fundamental aspect for understanding the relationship between our bacteria and oncological therapy, namely bidirectionality. In fact, it is now evident how these aspects influence each other, and one is the direct consequence of the other (Figure 1). In detail, the various microbial compositions can influence the response to chemotherapy by increasing or decreasing its effectiveness while, on the other hand, chemotherapy is able to modify the bacterial populations by producing a perturbation of their crucial balance (dysbiosis) and generating local alterations which could expose us more easily to toxic effects of drugs such as mucositis, as described in detail later.

## Impact of the microbiota on the effectiveness of chemotherapy treatments

**FIGURE 2:**
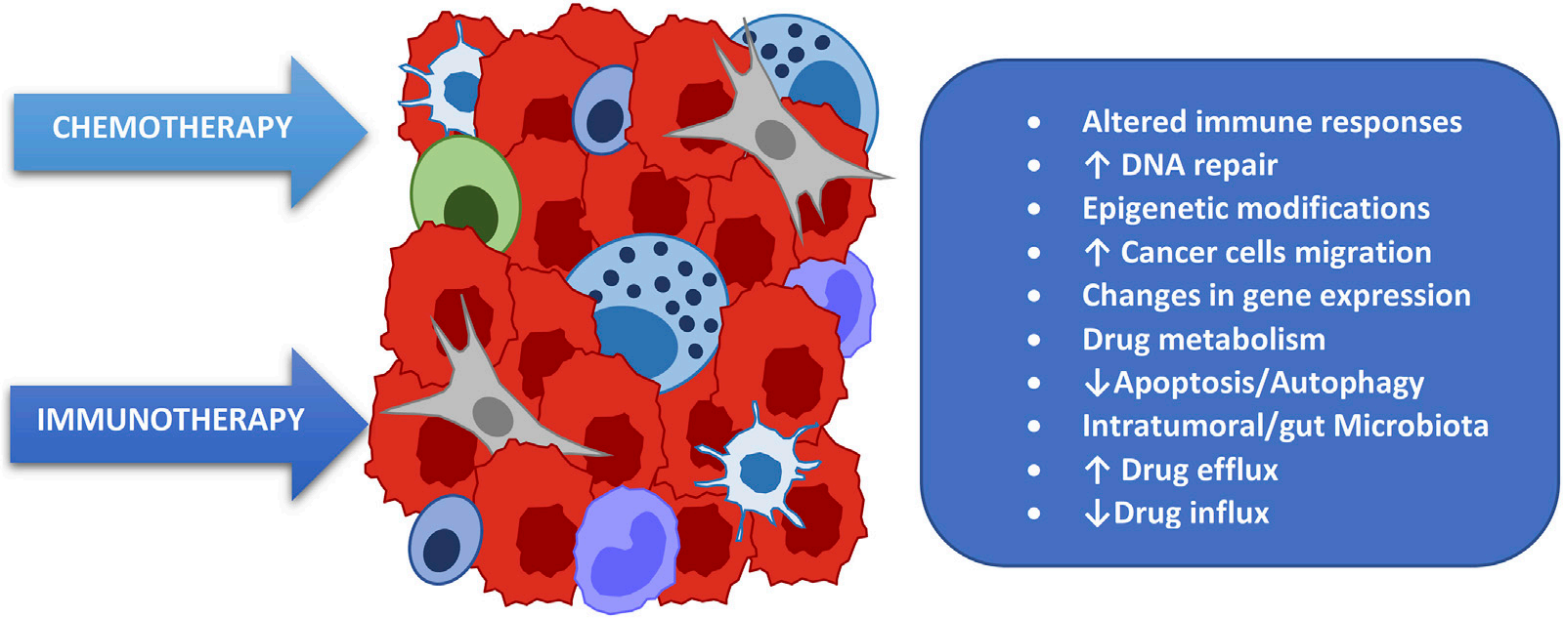
Main mechanisms related to the resistance of cancer cells to oncological treatments.

We have just introduced the concept of how the microbiota influences the response to chemotherapy treatments by modulating their efficacy and therefore the therapeutic response. The first mechanism to understand, in order to hypothesize an intervention capable of positively exploiting this fact, is the main way in which the microbiota alters the response to chemotherapy, namely through the induction of chemoresistance [6]. There are many mechanisms of interaction between chemotherapy and microbiota involved in this phenomenon, summarized in (Figure 2). Among the main ones we remember how the microbiota influences the functionality of the trans-membrane pump which regulates the amount of cytotoxic drug that enters the cell as well as that which is expelled, thus regulating the amount of drug that remains inside the tumor cell and hence the therapeutic potency [7]. A further and important mechanism is the reparative capacity of Deoxyribonucleic acid (DNA) induced by the microbiota [8]; although perhaps the most important mechanism associated with chemoresistance is the capacity of some bacterial strains to modulate, transform, change, and metabolize the chemotherapy itself, impacting on its efficacy [9]. To understand how much these and other mechanisms are able to influence the treatment characteristics and therefore how essential it is to know them and, when possible, to modulate them, we will examine some well-known clinical examples with results published in authoritative scientific journals.

### Microbiota-induced resistance to gemcitabine

**FIGURE 3:**
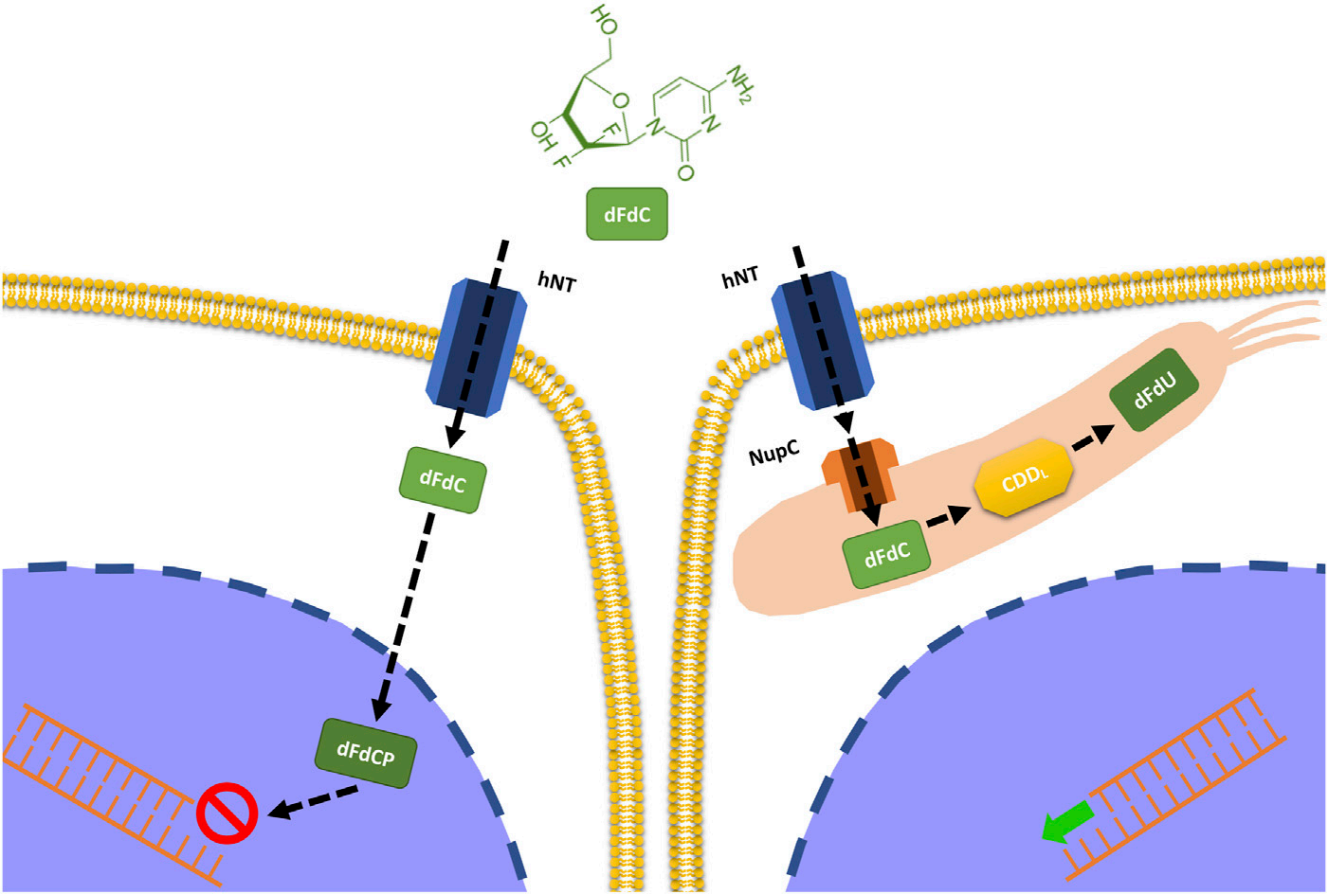
Impact of intratumoral bacteria on the metabolization of gemcitabine. Some bacteria (proteobacteria) have the ability to express the enzyme cytidine deaminase responsible for the inactivation of gemcitabine and therefore for the reduction of its efficacy.

Gemcitabine is known to be an antineoplastic drug of the group of specific pyrimidine cell cycle antimetabolites and is used for the treatment of numerous pathologies such as pancreas, lung, breast, bladder, and ovary cancers, as well as in many sarcomas, alone or in combination with other chemotherapy. Its anticancer activity is the result of the balance between its activation and inactivation. That is, it does not act in the injected form but is metabolized through some kinases into its active metabolites which are gemcitabine monophosphate, diphosphate, and triphosphate. Once these metabolites have performed the required cytotoxic action, they are degraded by other enzymes and in particular by cytidine deaminase which renders them inactive and ready for elimination [10]. However, in the presence of a particular microbial composition and more precisely in the case of an abundance of proteobacteria (gammaproteobacteria) this scenario is altered as these microbes are natural producers of the enzyme cytidine deaminase and therefore enhancers of the inactivation effect of this drug, leading ultimately to a reduction of its therapeutic capacity [11] (Figure 3). In this regard, a study published in Science in 2017 [12] shows precisely how gemcitabine chemotherapy treatment resistance in patients with pancreatic cancer is more present in those with a microbiota abnormally rich in proteobacteria and therefore highlights how a restoration of microbial eubiosis, in particular with a decrease of proteobacteria obtained with a specific antibiotic therapy, has increased the therapeutic response and therefore the improvement of prognosis in these subjects.

### Resistance to chemotherapy for *Fusobacterium nucleatum* -induced colorectal cancer

A further and effective example of the phenomenon of microbiota-related chemoresistance is related to the presence of *Fusobacterium nucleatum* (FN) and the efficacy of chemotherapy for colorectal cancer. In fact, it is now known and accepted that this bacterium, a commensal of the oral cavity responsible for numerous periodontal diseases, is also strongly implicated in the onset of colorectal cancer through inflammatory, genetic, and immune mechanisms [13]. What is less known is the ability of this bacterium not only to influence the onset of the disease but also to impact its progression and prognosis given its ability to increase autophagy. This cellular property is obviously positive for the repair of the numerous damages that our cells undergo on a daily basis but becomes counterproductive if the cellular damage is the goal of a pharmacological treatment [14]. This is the case with chemotherapy which normally acts precisely through cytotoxic damage to the cell, and it is clear that any reparative mechanism limits the effectiveness of the treatment. FN is counterproductive and therefore harmful in this sense precisely because of its property of inducing and increasing autophagy and therefore its presence ends up inducing chemo-resistance. Also in this case, there is an authoritative study [15] published in Cell in 2017 which highlights how patients with an abundant presence of FN were much more resistant to chemotherapy and therefore had a worse prognosis than those with a more favorable microbial structure.

### Increased efficacy of cyclophosphamide induced by microbiota

Microbial effect on chemotherapy acts not only in the sense of resistance, therefore reducing therapy effects and worsening the prognosis, but also in a positive way increasing the effectiveness of some chemotherapy and therefore opening the possibility of enhancing our oncological treatments through the modulation of the microbiota. This is the case, for example, with cyclophosphamide. It is an alkylating chemotherapeutic agent that acts in a cytostatic sense and is used for many oncological pathologies such as hematological ones and also many solid ones such as in neuroblastoma, some sarcomas, and breast and ovarian cancers [16]. Its antitumoral property is wide and at least in part due to its ability to stimulate and increase the antitumoral immune response [17]. Recent authoritative studies [18, 19] have shown how once administered it is able to select some bacterial populations within the lymphoid organs, thus determining an increase in the production of immune cells directed against tumor antigens.

## Impact of microbiota on the toxicity of chemotherapy treatments

As anticipated, the relationship between microbiota and chemotherapy treatments is not only related to the negative or positive modulation of pharmacological efficacy but is also closely linked to the possibility or not of augmenting or limiting side effects, in particular—but not only—the early ones. In fact, we have already seen how chemotherapy can alter the microbiota and how dysbiosis can be, in various ways, the cause or contributory cause of various side effects.

### Action of B-glucuronidase on the toxicity of irinotecan

**FIGURE 4:**
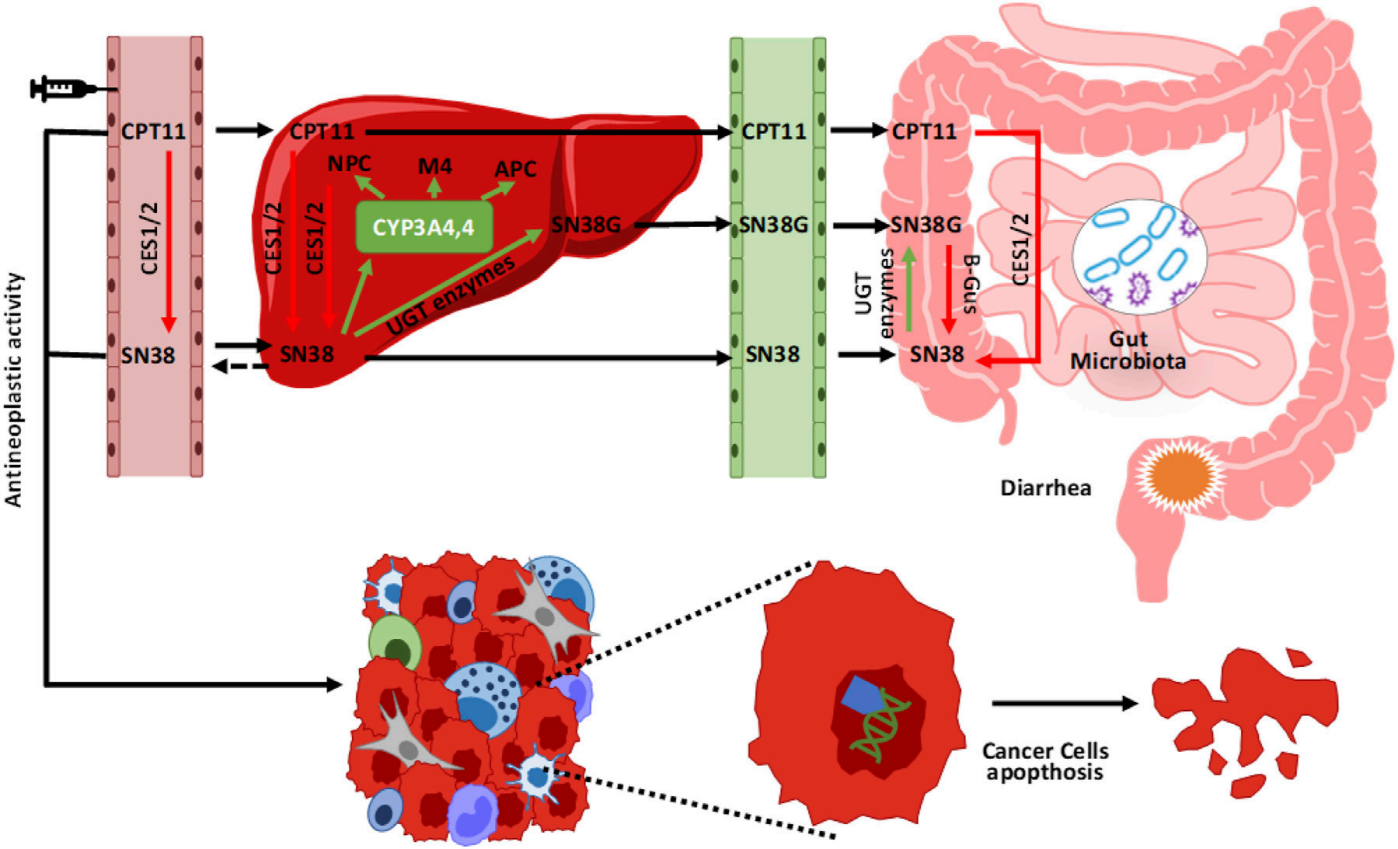
Metabolism and elimination of irinotecan (CPT11). Once injected, irinotecan is activated by some esterases (CES 1/2) into its active metabolite (SN38). After its anticancer action, it is again conjugated in the liver into an inactive compound ready to be eliminated (SN38G) mainly through the intestine and faeces. In its colonic passage, the presence of bacterial groups with enzymatic b-glucuronidase activity, reactivate the compound (SN38) again, effectively increasing its concentration and the risk of associated toxicity (e.g., mucositis).

One of the best documented examples of the action of bacteria on chemotherapy toxicity concerns the use of irinotecan. This chemotherapy, which belongs to the camptothecin class, is mainly used in colorectal cancer, non-small cell lung cancer, and ovarian cancer treatment [20]. As with other compounds, this one not does not act directly but only after its transformation into an active metabolite. In fact, it is a pro-drug and only after the action of some esterases is it transformed into its active metabolite called SN38, a pro apoptotic molecule that inhibits the enzyme Topoisomerase I with consequent cell inability to repair the damaged DNA. SN38 compound excretion with the bile and then with the feces takes place only after its passage through the liver with conjugation to SN38G [21]. However, during intestinal transit, the inactive compound interacts with the microbiota and within certain microbial conditions, such as the presence of abundant bacterial groups with b-glucuronidase activity (i.e. capable of producing an enzyme called b-glucuronidase). It is de-conjugated and made available for reabsorption as the active molecule, effectively increasing its concentration and consequently its toxicity, for example by generating intestinal damage such as mucositis Figure 4 [22].

### Influence of the microbiota on the appearance of chemotherapy mucositis

With regard to chemotherapy-induced mucositis, the example just described concerning Irinotecan and its relationship with bacterial activities such as beta-glucuronidase production represents only one of the links between the microbiota and the appearance or reduction of this fundamental side effect, perhaps the main factor responsible for decreased compliance and consequent therapeutic failure. Mucositis is a complication of various cancer therapies such as radiotherapy and chemotherapy [23]. It is characterized by very disabling symptoms such as presence of nausea, abdominal cramps, swelling, and above all diarrhea, even to a high degree [24]. It is supported by inflammatory and apoptotic phenomena and ultimately related to the intestinal microbial composition. Sonis [25] in 2004 described a mechanism of onset divided into 5 stages considering the microbial infection to be only a consequence of the inflammatory process triggered by the direct action of chemotherapy on the enterocyte wall. Recently, however, new research has highlighted a much more important and above all causal role of the microbial structure on the genesis of mucositis. In practice, the microbiota would be a link between the potential toxicity of the drug and the effective initiation of the chain of events that lead to mucositis. The presence or rather the absence of some bacterial groups (in particular those capable of producing short-chain fatty acids or SCFAs, especially butyrate) generates a microenvironment which favors both directly (through the stimulation of particular receptors called TLR4) and indirectly (producing an increase in bacterial permeability and therefore the translocation of GRAM-bacteria and therefore of lipopolysaccharide—LPS in the circulation) the genesis of inflammatory and immune processes responsible for the toxic effects [26]. The main bacterial populations linked with effectiveness and toxicity of chemotherapy are summarized in Table 1.

**TABLE 1:** Examples of bacterial populations active on CHT efficacy and toxicity.

Efficacy	
Gammaproteobacteria	Gemcitabine efficacy reduction through early inactivation
* Fusobacterium*	Increased resistance to many chemotherapeutics through autophagy modulation
GRAM+ (some induced by cyclophosphamide)	Increase of therapy efficacy through an immunity stimulation
Toxicity	
Firmicutes/Bacteroidetes	Increase of Irinotecan toxicity through Beta-glucuronidase activity increase
↓ Bacteria SCFAs Producers	Increased toxicity of some chemotherapy (mucositis)

## Possible interventions

Considering the described relationship between microbial composition and influence over efficacy and toxicity of various chemotherapies used in various forms of cancer, it appears quite intuitive and immediate how an intervention to modulate and shape an “ideal or favorable” microbiota could allow us to improve efficacy, tolerability, compliance, and outcome of oncological therapies. The use of prebiotics, probiotics and nutraceuticals is becoming increasingly central in accompanying the various chemotherapy regimens as a result of the increasingly frequent publication of important scientific data supporting this hypothesis. In particular, considering chemotherapy and the mechanisms we have just described, it is increasingly evident that one of the potentially most effective and easiest ways to intervene is modulating the microbiota in a eubiotic sense. For example, it appears convenient to enhance microbiota production of short-chain fatty acids (SCFAs), using prebiotics that nourish and promote the growth of the bacteria responsible for their production [27], or directly assuming probiotics, i.e., bacterial groups with suitable characteristics of engraftment and vitality, capable of ensuring the production of adequate quantities of SCFAs and in particular of butyric acid [28]. It is also important to underline that on the market there are already formulations suitable for clinical use supported by abundant scientific literature. The *Clostridium Butyricum Miyairi* CBM588 strain marketed in Italy as Butirrisan © is the best-known example [29].

### Butyric acid in the modulation of chemotherapy efficacy and toxicity

**FIGURE 5:**
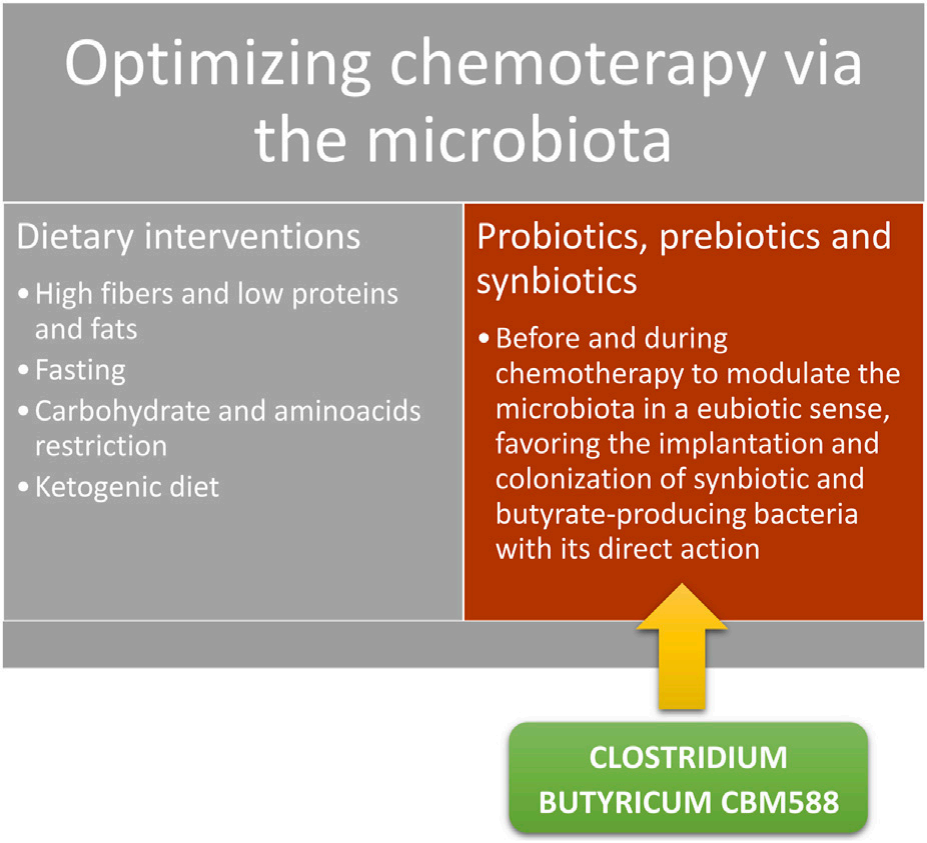
Possible interventions to increase the proportion of SCFAs in particular of butyrate and modulate the toxicity and efficacy of chemotherapy.

Short-chain fatty acids (acetate, propionate, and butyrate) are a class of saturated fatty acids with an aliphatic chain consisting of fewer than 6 carbon atoms. They are mainly produced during the fermentation of fibers (undigested carbohydrates in the small intestine, fructo-oligosaccharides, pectin, inulin, etc.) operated precisely by particular bacterial classes of the intestinal microbiota [30]. These fermentative products are essential for a whole series of pathophysiological processes. For example, they are the source of most of the enterocytes’ energy, so much so that they are crucial for colonic wall health and stability; therefore their depletion should be avoided in order to prevent inflammatory bowel diseases [31]. Furthermore, their link with oncological diseases has recently been highlighted, as SCFA have the recognized ability to prevent development of cancer (especially—but not only—colon cancer) [32] and could influence conventional oncological treatments effectiveness [33]. In this scenario, the butyric acid produced by a particular bacterial class called *Clostridium Butyricum* (CB) of the Clostridiales group seems to have (at least for now), supported by clinical data, a decidedly more decisive role than other SCFAs. Over the last few years, it has shown unequivocal properties both in modulation of efficacy and toxicity of oncological therapies (in particular chemotherapy and immunotherapy) [34]. One of the main and recent examples of the ability of this compound to impact chemotherapy toxicity is the one published by [35] in 2019 related to doxorubicin cardiotoxicity. In this work it is clearly demonstrated that butyrate use is related to a reduced left ventricular dilatation, fibrosis, and cardiomyocytes apoptosis induced by chemotherapy. The study also highlights a series of effects on some cardiotoxicity biomarkers such as mitochondrial respiration and reactive oxygen substances (ROS) production, confirming the ability of the compound to improve cardiac tolerability to the potential toxicity induced by anthracyclines and doxorubicin in particular. Another indirect anti-toxic effect of this bacterial metabolite is the intestinal wall health improvement and inflammatory phenomena reduction already observed as potential treatment of chemotherapy related mucositis (though not only). This effect is also well documented in literature [36] where it is demonstrated that its administration reduces taxanes intestinal toxicity, such as paclitaxel (one of the most widely used chemotherapy in case of ovarian, breast, and lung cancers among others), precisely by protecting and safeguarding intestinal wall health, decreasing chemotherapy induced dysbiosis and permeability of the wall itself. A recent placebo-controlled study performed in China [37] examined the effect of CB on patients receiving platinum-based chemotherapy plus anti-angiogenics for non-squamous non-small cell lung cancer. This study confirmed a significant reduction in drug toxicity in the group receiving CB compared to a placebo. Furthermore, a eubiotic effect of the microbiota is highlighted with an increase also of bifidobacteria and lactobacilli, as well as a not statistically significant but still evident effect on the progression free survival (PFS) and overall survival (OS) of these patients. However, this significance has been amply demonstrated in a Japanese study [38] in which patients subjected in this case to immunotherapy treatment for lung cancer improved their PFS and OS compared to the placebo group even after antibiotic therapy. In conclusion we cannot fail to mention the work of [39] which, as an example of butyrate-dependent chemotherapy efficacy improvement, allows us to highlight the ability of CB to increase the apoptotic capacity of 5-fluoruracil (5-FU) in colorectal cancer through its (limiting) action on glucose transport and aerobic glycolysis, limiting energy source of tumor cells in metabolic reprogramming or Warburg effect [40]. Therefore, in consideration of what has been exposed, CB seems to be an important weapon at our disposal (immediately) to try to modulate chemotherapy toxicity and efficacy and therefore the quality of life and prognosis of our patients. The methods of intervention can be summarized in the following diagram (Figure 5).

### Possibility of using other compounds. The example of berberine and curcumin

Although the butyrate approach is apparently the most documented, other non-direct bacterial interventions have shown good modulation capacity in oncological treatment scenarios, so much so that they can be taken into account both alone and in association with the therapies just described. This is the case of some nutraceuticals or herbs that are now widely discussed and studied, such as berberine (BBR) [41] and curcumin (CUR) [42]. In fact, both of these compounds have already shown unequivocal properties of toxicity reduction and/or tumor cells sensitization to chemotherapeutics. For example, BBR is one of the compounds that seem able to modify the effect of the toxicity of Irinotecan already described above. In fact, being a natural inhibitor of the enzyme b-glucuronidase, BBR reduces the deconjugation action of this enzyme, reducing the possibility of reactivation of the inactive metabolite of irinotecan, consequently reducing its toxic effect (mucositis). This possibility is well documented in a 2021 work concerning colorectal tumors [43] and where the protective capacity of intestinal permeability and consequently anti-inflammatory of BBR is highlighted precisely through the modulation of the deconjugating enzyme and therefore the reduction of side effects of treatment. Also, BBR could be considered as an excellent alternative in terms of raising awareness of oncological treatments. As we have previously highlighted, one of the mechanisms of resistance to oncological drugs is that of autophagy which makes tumor cells less and less sensitive to the toxic action of chemotherapy, effectively establishing a sort of worsening resistance to the treatment. Being a natural inhibitor of this mechanism, BBR appears to be a potential ally to the action of chemotherapy to mitigate the problem of resistance and therefore increasing its sensitivity and in fact its effectiveness [44].

Furthermore, BBR does not seem to be the only herb capable of modulating the autophagy action of tumor cells, improving the efficiency of oncological treatments. For example, CUR, as demonstrated by many works, also appears effective in this important anticancer action. Indeed, the CUR, in addition to also having important properties of reducing chemotherapy (and radiotherapy) side effects [45], has evident chemosensitization capacities and therefore seems capable of increasing the action of some chemotherapeutics [46].

In addition to the aforementioned effect on autophagy, we recall that one of the most important mechanisms in the onset of the chemo-resistance phenomenon is determined by the activity of an extrusion pump on the cell surface managed by some proteins, the most important of which is glycoprotein P which eliminates the drug from the cell making the treatment less and less effective [47]. CUR is a potent natural inhibitor of P-glycoprotein and its administration has already demonstrated an increase in cellular sensitivity to chemotherapy precisely thanks to the inhibition of this limiting cellular factor [48]. Some works have already highlighted the effectiveness of this treatment, including that of [49] which demonstrates the increased sensitivity of breast cancer cells to the action of doxorubicin when treated with CUR. Equal positive results are also already evident for other types of cancer such as that of the cervix and ovary [50, 51], or therapy, for example with anti-estrogens such as tamoxifen [52].

## Conclusion

Oncological therapies and in particular their clinical efficacy and tolerability are influenced by many factors, both pharmacological and individual of the patients themselves. Among these, the importance of the microbiota (colonial in particular but also of the various districts involved) is increasingly evident and impactful. In the coming years, inevitably, oncological treatments will need to be accompanied by in-depth microbial analyses and improved by the microbial parameters (biodiversity, eubiosis, metabolic capacity, etc.) in order to favor an environment conducive to the action of the drugs and/or the reduction of their toxicity. Chemotherapy is certainly one of the treatments most closely related to the microbiota and numerous studies are highlighting how certain bacterial characteristics can favor or disadvantage the therapeutic result and how modulation through the use of prebiotics, probiotics, and nutraceuticals is and will be increasingly decisive in managing such a scenario. This review—focused in particular on chemotherapy—has highlighted some examples in this sense, emphasizing the importance of some interventions such as that of the CB of the BBR and the CUR but also opening up the possibility of studying other compounds in order to further improve the effectiveness of oncological treatments.
